# Risk of Respiratory Disease in people with Bipolar Disorder: A Systematic Review and Meta-Analysis.

**DOI:** 10.1192/j.eurpsy.2025.585

**Published:** 2025-08-26

**Authors:** A. Jimenez, D. Laguna, M. I. Alarcón, M. Reyes, M. J. Jaén, F. Sarramea

**Affiliations:** 1 Hospital Univeristario Reina Sofía; 2 IMIBIC; 3 Univesity of Córdoba, Córdoba, Spain

## Abstract

**Introduction:**

People with bipolar disorder (BD) have an increased risk of premature mortality, and the respiratory mortality rate is higher than those of the general population. However, the evidence on respiratory disease in this population has not been meta-analyzed.

**Objectives:**

To systematically review and meta-analyze the frequency of respiratory diseases in patients with BD and to compare prevalence and Odds Ratio (OR) with the general population.

**Methods:**

A systematic literature search was conducted in Pubmed, PsycINFO, Scielo and Scopus from inception to June 2, 2023, and a snowball search of reference and citation lists was conducted. Inclusion criteria were studies reporting diagnoses of respiratory diseases (asthma, chronic obstructive pulmonary disease (COPD), pneumonia, lung cancer and tuberculosis) in people with BD according to operationalized criteria and where possible, control group. This study followed Preferred Reporting Items for Systematic Reviews and Meta-analyses (PRISMA) and MOOSE reporting guidelines. A pair of reviewers independently extracted data using a predefined data extraction form and a senior co-author was consulted in cases of disagreement. The risk of bias and methodological quality was assessed using the adapted Newcastle-Ottawa scale.

**Results:**

Of the 2,158 articles screened, 20 including 962,352 people with BD and 37,340,405 control group, met the inclusion criteria (see Figure 1). Prevalence and OR of respiratory disease in people with BD was the main outcome as percentage point estimates with corresponding 95% CIs. In people with BD, the prevalence of COPD was 9.14% (95%CI: 6.61%-12.5%), asthma 6.4% (95%CI: 4.56%-8.91%), pneumonia 2.78% (95%CI: 2.51%-3.08%) and lung cancer 0.44% (95%CI:0.23%-0.84%) (see Figure 2). Compared to the general population (see Figure 3), people with BD had significantly higher rates of COPD (OR: 1.73; 95% CI: 1.40-2.14), showing an increased rate in younger and female patients; asthma (OR: 1.91, 95% CI: 1.25-2.94), with a greater rate in younger patients; and pneumonia (OR: 2.82, 95% CI: 1.33-5.99).

**Image 1:**

**Figure:**
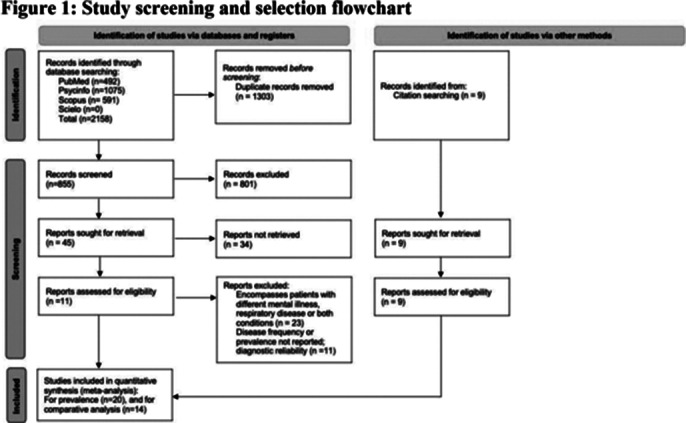

**Image 2:**

**Figure:**
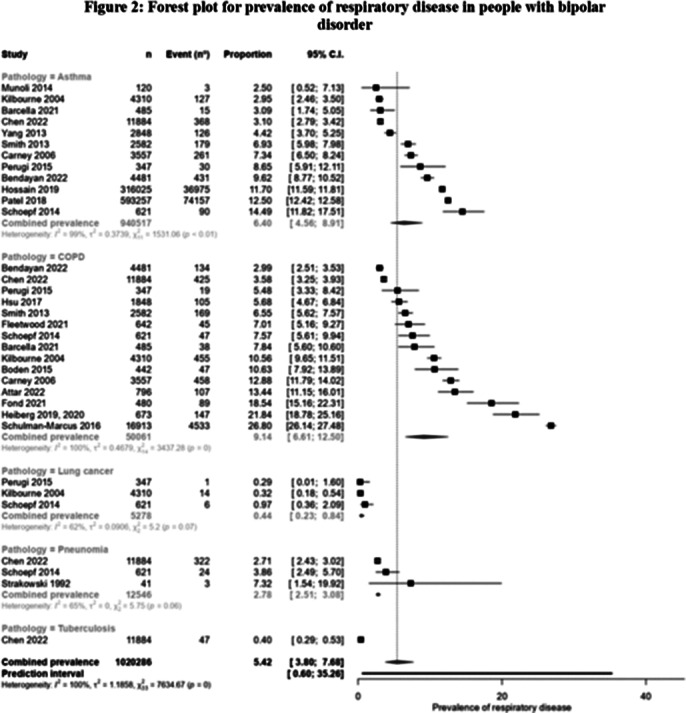

**Image 3:**

**Figure:**
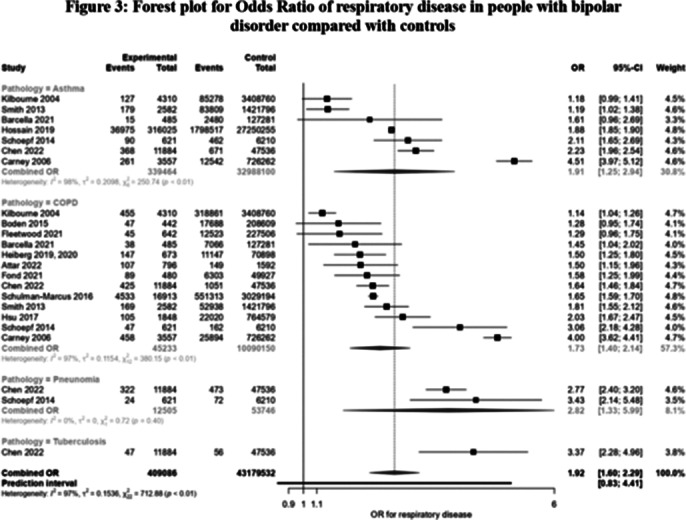

**Conclusions:**

In the first meta-analysis on the topic, BD was associated with an increased risk of respiratory illness versus the general population. In COPD and asthma, young people and women are at particular risk. Prevention programs are urgently needed.

**Disclosure of Interest:**

None Declared

